# Supplementary material for: An Interplay between Mitochondrial and ER Targeting of a Bacterial Signal Peptide in Plants
Source: Plants (Basel). 2023 Jan 31;12(3):617. doi: 10.3390/plants12030617 (PMC9920398; doi:10.3390/plants12030617)
Supplement: Supplementary file 1 [file plants-12-00617-s001.zip › plants-2113996-supplementary.pptx]

## Slide 1
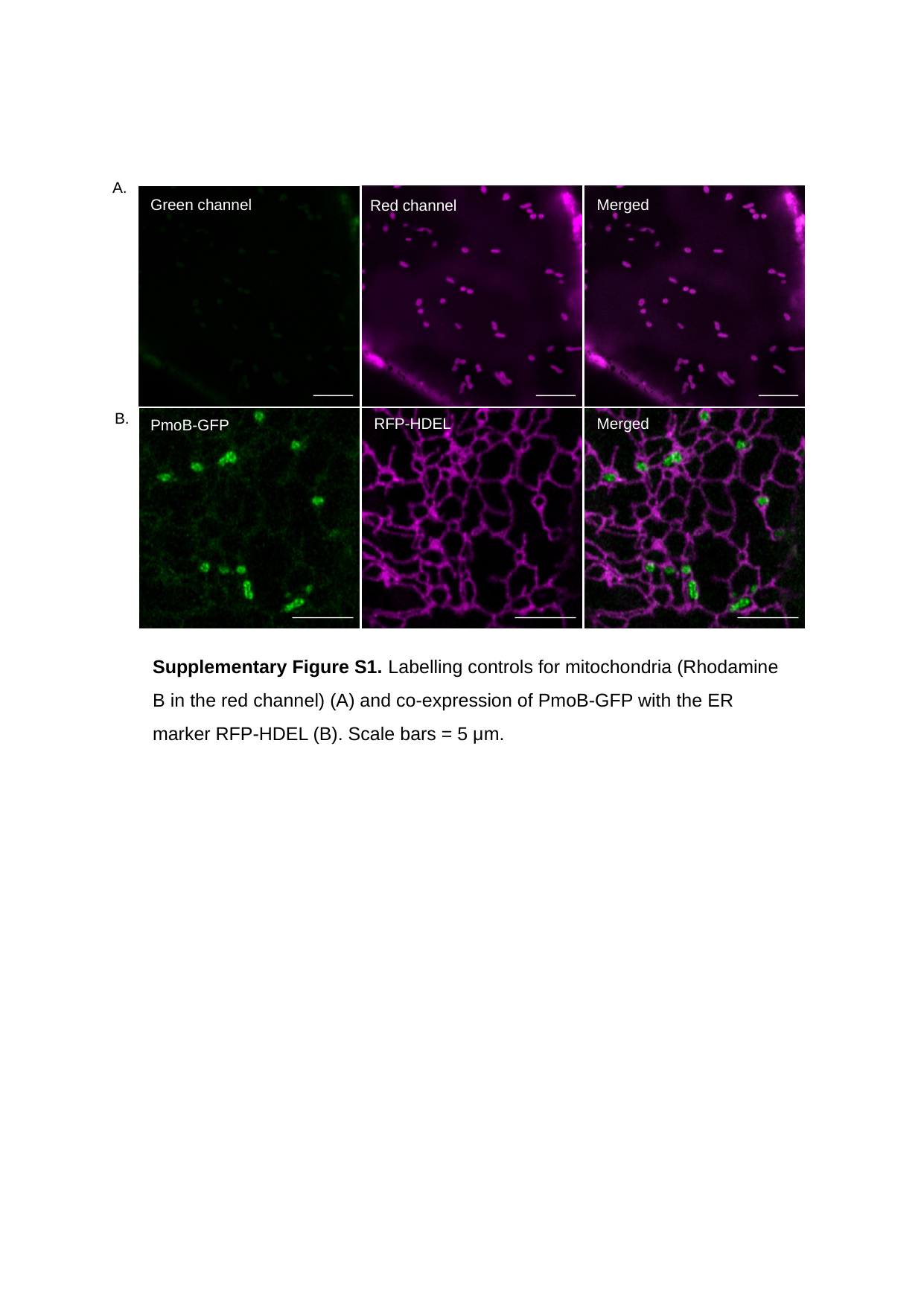

A.
Merged
Green channel
Red channel
B.
Merged
RFP-HDEL
PmoB-GFP
Supplementary Figure S1. Labelling controls for mitochondria (Rhodamine B in the red channel) (A) and co-expression of PmoB-GFP with the ER marker RFP-HDEL (B). Scale bars = 5 μm.

## Slide 2
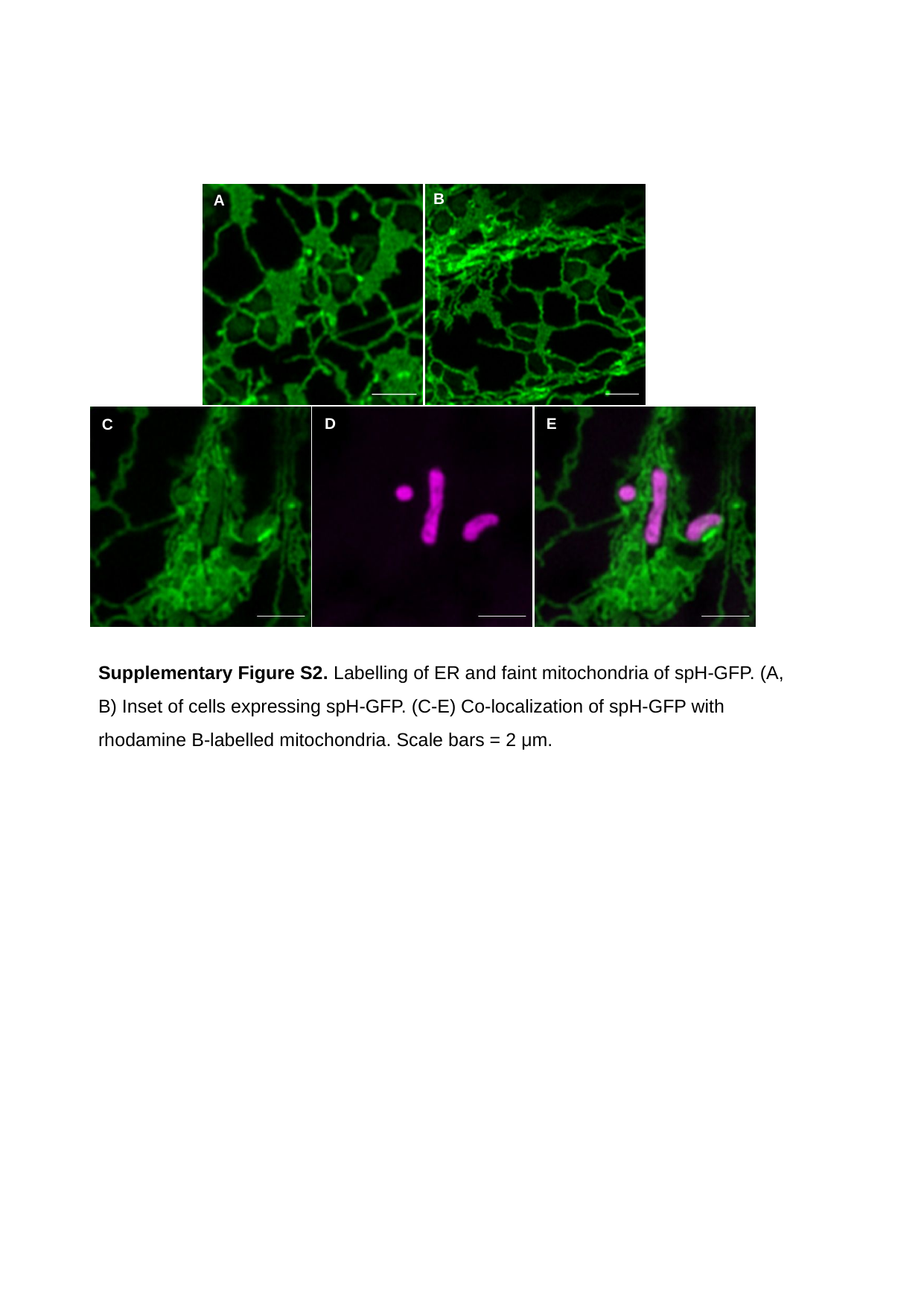

B
A
E
D
C
Supplementary Figure S2. Labelling of ER and faint mitochondria of spH-GFP. (A, B) Inset of cells expressing spH-GFP. (C-E) Co-localization of spH-GFP with rhodamine B-labelled mitochondria. Scale bars = 2 μm.

## Slide 3
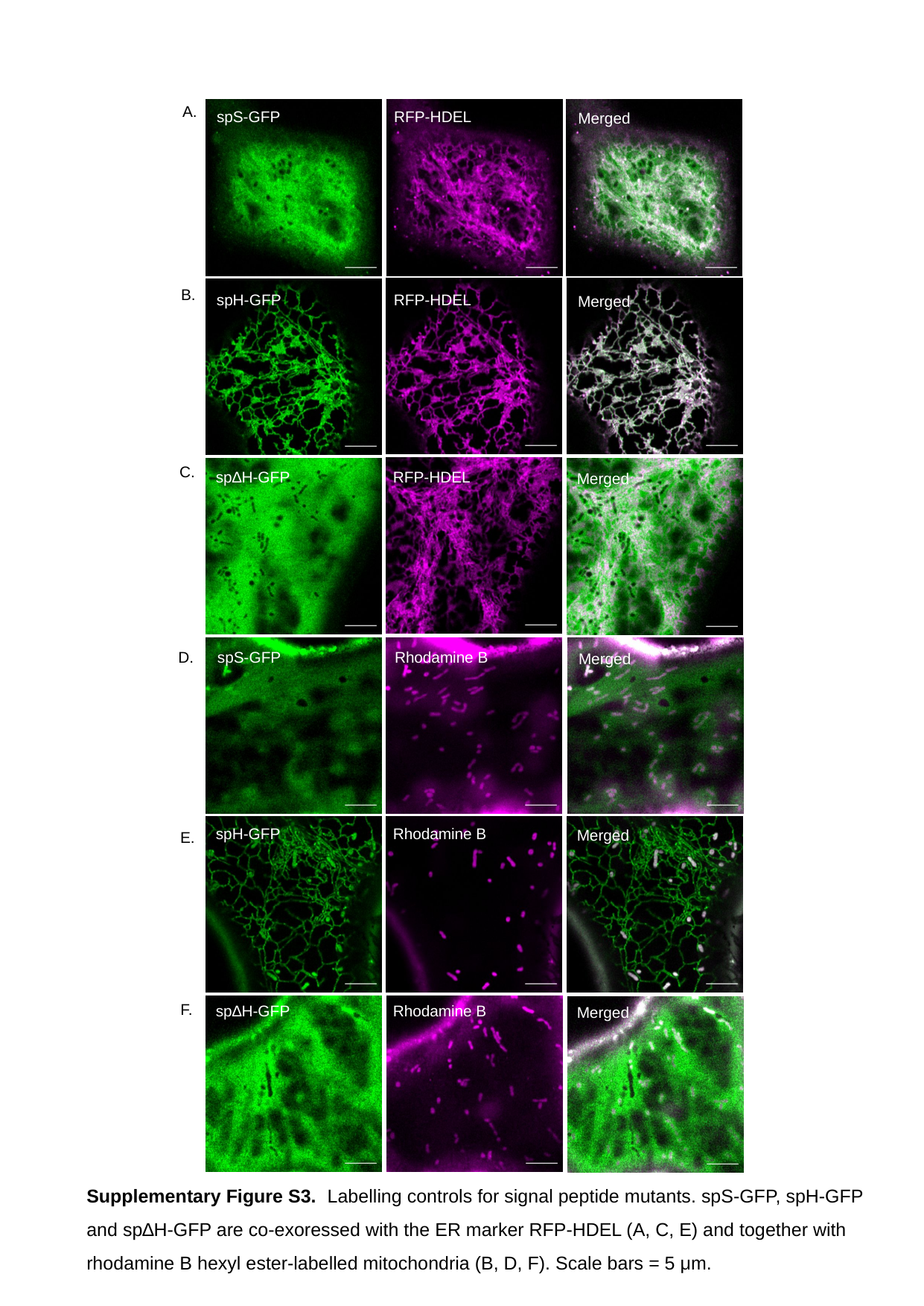

A.
spS-GFP
RFP-HDEL
Merged
B.
spH-GFP
RFP-HDEL
Merged
C.
sp∆H-GFP
RFP-HDEL
Merged
D.
spS-GFP
Rhodamine B
Merged
spH-GFP
Rhodamine B
Merged
E.
F.
sp∆H-GFP
Rhodamine B
Merged
Supplementary Figure S3. Labelling controls for signal peptide mutants. spS-GFP, spH-GFP and sp∆H-GFP are co-exoressed with the ER marker RFP-HDEL (A, C, E) and together with rhodamine B hexyl ester-labelled mitochondria (B, D, F). Scale bars = 5 μm.

## Slide 4
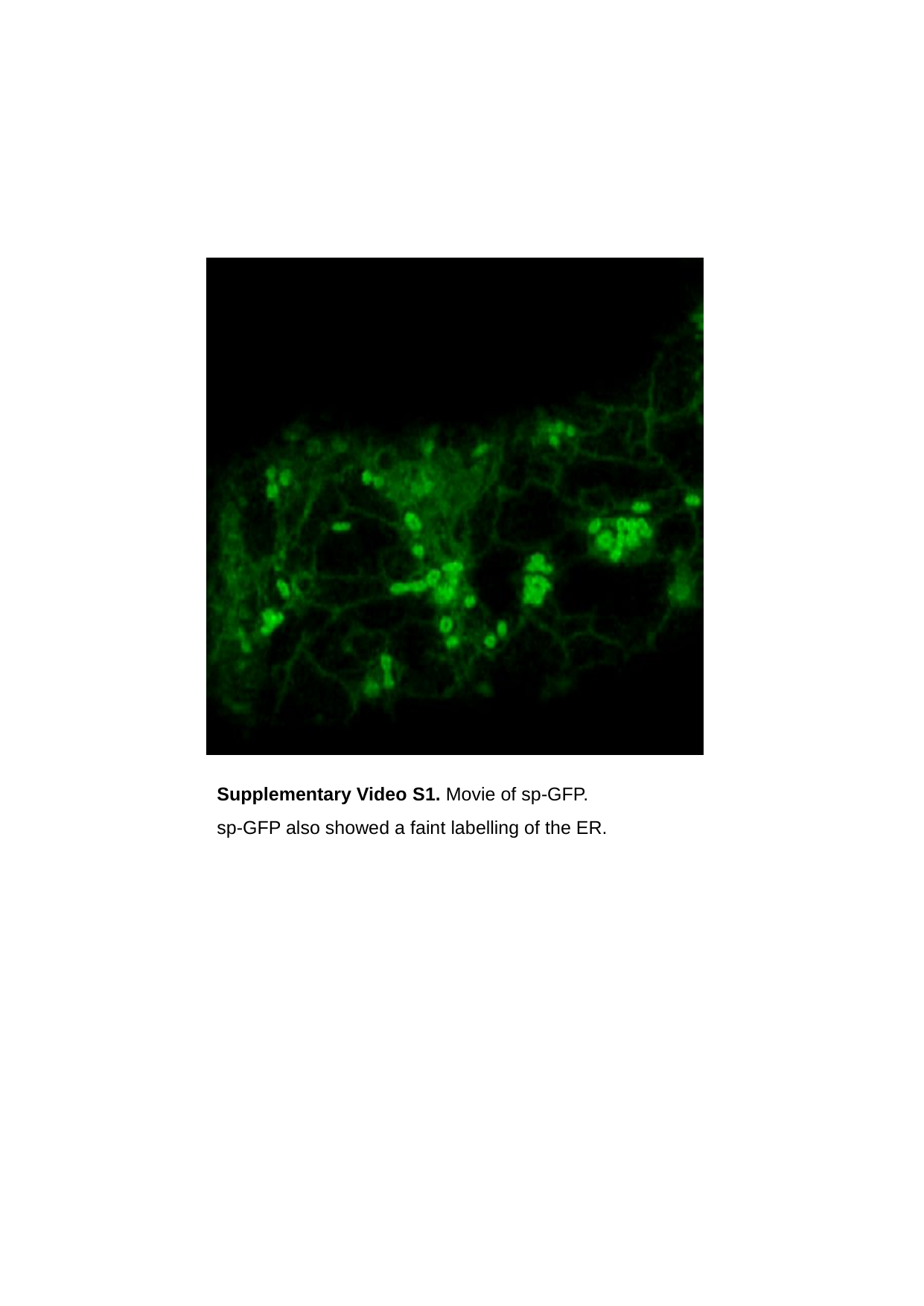

Supplementary Video S1. Movie of sp-GFP.
sp-GFP also showed a faint labelling of the ER.

## Slide 5
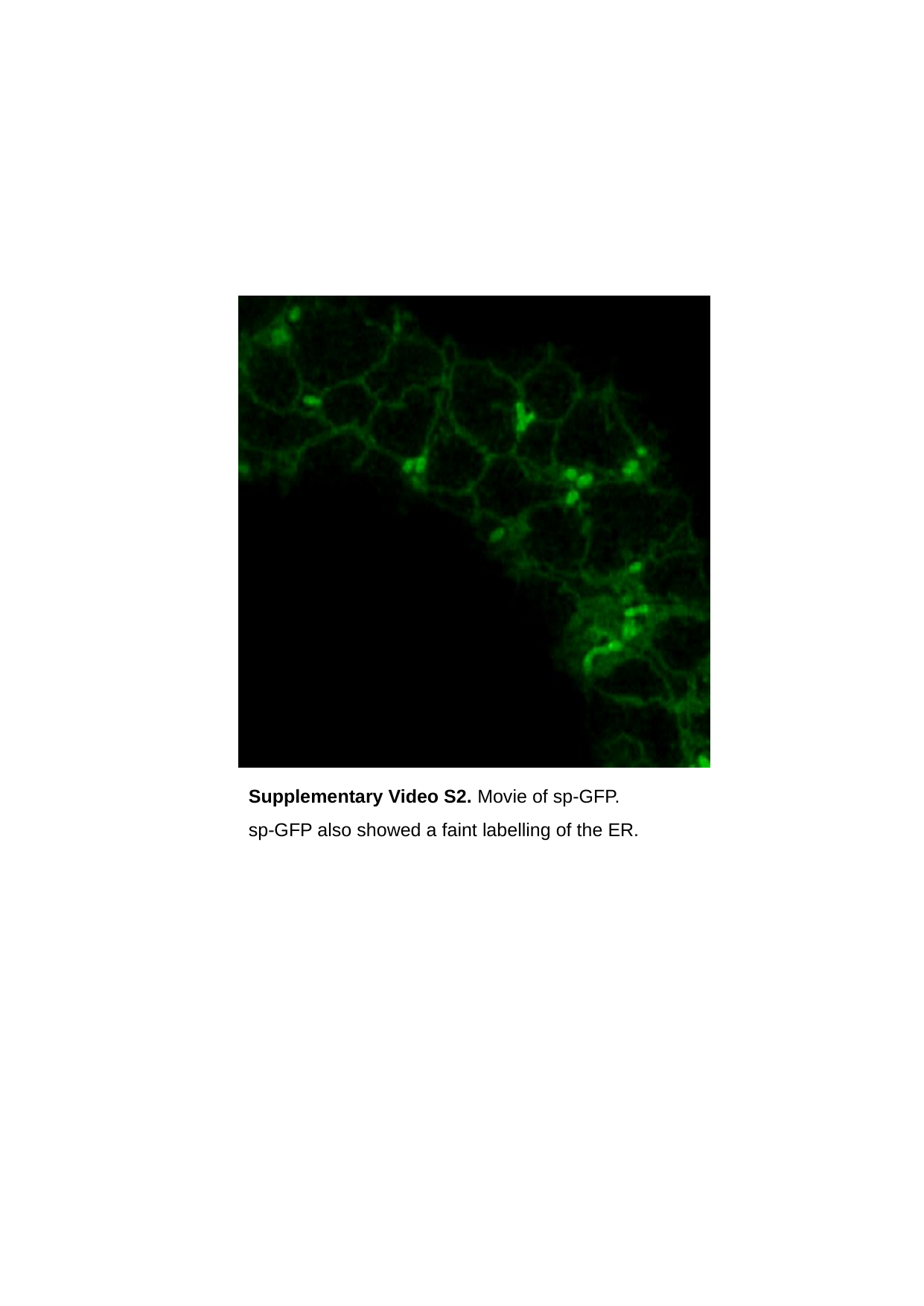

Supplementary Video S2. Movie of sp-GFP.
sp-GFP also showed a faint labelling of the ER.
